# Analysing connectivity with Granger causality and dynamic causal modelling

**DOI:** 10.1016/j.conb.2012.11.010

**Published:** 2013-04

**Authors:** Karl Friston, Rosalyn Moran, Anil K Seth

**Affiliations:** 1 The Wellcome Trust Centre for Neuroimaging, University College London, Queen Square, London WC1N 3BG, UK; 2 Sackler Centre for Consciousness Science and Department of Informatics, University of Sussex, Brighton BN1 9QJ, UK

## Abstract

This review considers state-of-the-art analyses of functional integration in neuronal macrocircuits. We focus on detecting and estimating *directed connectivity* in neuronal networks using *Granger causality* (GC) and *dynamic causal modelling* (DCM). These approaches are considered in the context of functional segregation and integration and — within functional integration — the distinction between *functional* and *effective* connectivity. We review recent developments that have enjoyed a rapid uptake in the discovery and quantification of functional brain architectures. GC and DCM have distinct and complementary ambitions that are usefully considered in relation to the *detection* of functional connectivity and the *identification* of models of effective connectivity. We highlight the basic ideas upon which they are grounded, provide a comparative evaluation and point to some outstanding issues.

## Highlights

► A brief introduction to the analysis of directed connectivity in brain networks. ► An overview of advances in Granger causality and dynamic causal modelling. ► A comparative evaluation of both approaches in terms of their pros and cons.

**Current Opinion in Neurobiology** 2013, **23**:172–178

This review comes from a themed issue on **Macrocircuits**

Edited by **Steve Petersen** and **Wolf Singer**

For a complete overview see the Issue and the Editorial

Available online 21st December 2012

0959-4388/$ – see front matter, © 2012 Elsevier Ltd. All rights reserved.

**http://dx.doi.org/10.1016/j.conb.2012.11.010**

## Introduction

Several dichotomies have proved useful in thinking about analytic approaches to functional brain architectures. Perhaps the most fundamental is the distinction between *functional segregation* and *integration*. Functional segregation refers to the anatomical segregation of functionally specialised cortical and subcortical systems, while functional integration refers to the coordination and coupling of functionally segregated systems [1^••^]. Within functional integration, two main classes of connectivity have emerged — *functional* and *effective* connectivity. Functional connectivity refers to the statistical dependence or mutual information between two neuronal systems, while effective connectivity refers to the influence that one neural system exerts over another [2•, 3]. This distinction is particularly acute when considering the different analyses one might apply to electrophysiological or neuroimaging timeseries.

### Functional and effective connectivity

Because functional connectivity is defined in terms of statistical dependencies, it is an operational concept that underlies the detection of (inference about) a functional connection, without any commitment to how that connection was caused. In other words, one tests for dependencies between two or more timeseries, to reject the null hypothesis of statistical independence. This is equivalent to assessing the *mutual information* and testing for significant departures from zero. At its simplest, this involves assessing (patterns of) correlations — of the sort that define intrinsic brain networks. An important distinction — within functional connectivity — rests on whether dependencies are instantaneous or reflect an underlying dynamical process, in which causes precede consequences. This leads to the distinction between analyses of *directed* and *undirected* functional connectivity that do and do not appeal to temporal precedence respectively. Common examples of techniques used to assess undirected functional connectivity (dependencies) include independent components analysis [4] and various measures of synchrony, correlation, or coherence [5]. However, we will focus on analyses of directed functional connectivity — of which the prime example is Granger causality (GC) [6^•^]. This is because coupling in the brain is both directed and largely reciprocal (producing cyclic graphs or networks with loops that preclude structural causal modelling). As we will see below, GC and related concepts such as transfer entropy (TE) rest on establishing a statistical dependence between a local measurement of neuronal activity and measurements of activity elsewhere *in the past*.

**Figure 1:**
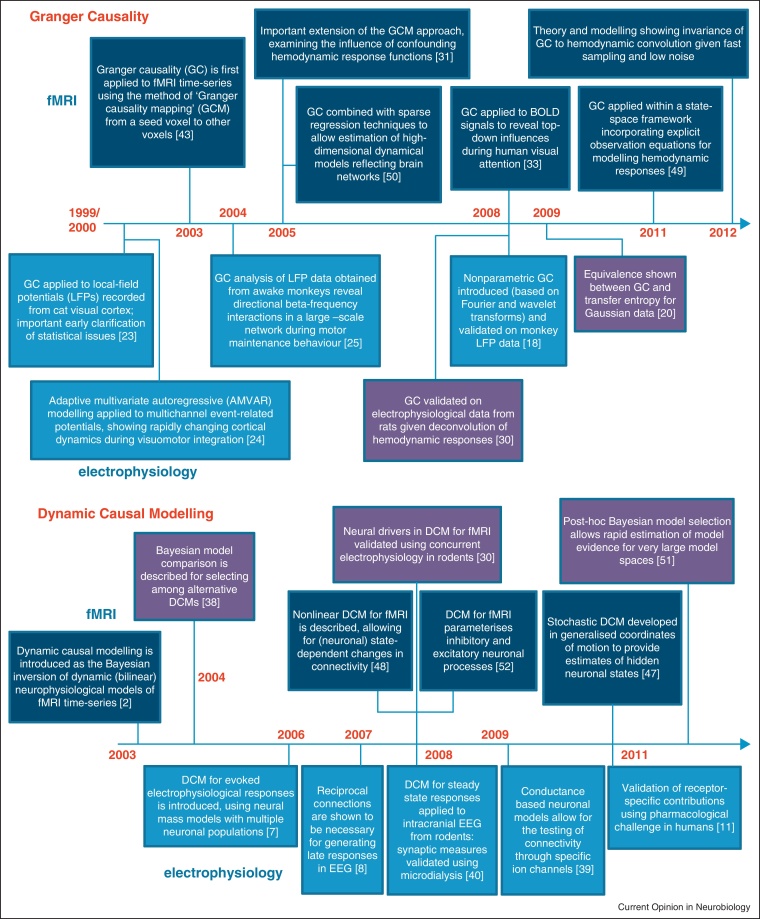
A timeline of recent advances in Granger causality (top panel) and dynamic causal modelling (bottom panel). Entries above the time lines pertain to functional magnetic resonance imaging (MRI) and those below the lines report specific developments for electrophysiology.

Functional connectivity considers dependencies between measured neurophysiological responses. In contrast, effective connectivity is between hidden neuronal states generating measurements. Crucially, effective connectivity is always directed and rests on an explicit (parameterised) model of causal influences — usually expressed in terms of difference (discrete time) or differential (continuous time) equations. The most popular approach to effective connectivity is dynamic causal modelling (DCM) [7, 8, 9, 10, 11••, 12••]. In this context, causality is inherent in the form of the model, where fluctuations in hidden neuronal states cause changes in others: for example, changes in postsynaptic potentials in one area are caused by inputs from other areas. The parameters of dynamic causal models correspond to effective connectivity — usually cast as synaptic density or coupling parameters — that are optimised by fitting the model to data. The notion of effective connectivity stems from the pioneering work of Gerstein and Perkel [13] in early attempts to interpret multivariate electrophysiological recordings. At its inception, effective connectivity referred to models; in the sense of the simplest possible circuit diagrams that explain observed responses [14]. In modern parlance, these correspond to dynamic causal models with the greatest evidence: namely, models with the minimum complexity that furnish an accurate explanation for data (see below). In what follows, we review recent developments in the analysis of directed functional connectivity with GC and TE, the analysis of directed effective connectivity with DCM and then consider the approaches in light of each other. Figure 1 provides an overview of recent developments in these techniques.

## Granger causality and transfer entropy

The core idea behind GC is that X ‘Granger causes’ Y if X contains information that helps predict the future of Y better than information already in the past of Y (and in the past of other ‘conditioning’ variables Z). The most common implementation of GC is via linear vector autoregressive (VAR) modelling of timeseries data, enabling both statistical significance testing and estimation of GC magnitudes [6•, 15•, 16]. However, GC is not limited to this implementation; it can use nonlinear, time-varying, and non-parametric models [17, 18]. In particular, TE [19] represents an information-theoretic generalisation of GC that does not require a parameterised model (is model-free). Specifically, the TE from X to Y is zero if, and only if, Y is conditionally independent of X's past, given its own past. Importantly, for Gaussian data, TE is equivalent to GC [20^••^], furnishing a useful interpretation of GC in terms of information transfer in ‘bits’. Related approaches include partial directed coherence and the directed transfer function; see [21] for a review. Here we focus on the most popular of these techniques, namely GC:

Following its introduction within econometrics [6•, 15•], GC has been applied in neuroscience partly because it is simple to estimate, given (stationary stochastic) timeseries. Such data are generated by a wide range of neuroimaging and neurophysiological methods. GC has some useful properties including a decomposition of causal influence by frequency [15^•^] and formulation in an ‘ensemble’ form, allowing evaluation of GC between multivariate sets of responses [22]. GC has provided useful descriptions of directed functional connectivity in many electrophysiological studies [23, 24, 25]. Recently, Bosman et al. [26^••^] analysed electrocorticographic data from macaque monkeys to show that ‘bottom-up’ signals across multiple cortical regions were most prominent in the gamma band, while ‘top down’ influences dominated at beta frequencies — a finding that is strikingly congruent with neural implementations of predictive coding [27]. GC can also be applied to standard EEG or MEG signals, either at the source or sensor level (following spatial filtering to reduce the impact of volume conduction). For example, Barrett et al. [28^•^] used source-localised EEG to show that gamma-band GC between posterior and anterior cingulate cortices reliably increased during anaesthetic loss of consciousness, extending previous results obtained using (undirected) phase synchrony [29]. We will turn to this example later in the context of DCM.

The application GC to fMRI is more controversial, given the slow dynamics and regional variability of the haemodynamic response to underlying neuronal activity [30, 31]; and see ‘Pros and Cons’ below. While naïve application of GC to fMRI data is unlikely to be informative, careful consideration of the methodological issues has permitted some useful applications that have produced testable hypotheses. For example, Wen et al. [32^•^] analysed fMRI data obtained from a cued spatial visual attention task; finding that GC from dorsal to ventral frontoparietal regions predicted enhanced performance, while GC in the reciprocal direction was associated with degraded performance. These findings are consistent with the notion that dorsal attentional regions mediate goal-oriented top-down deployment of attention, while ventral regions mediate stimulus-driven bottom-up reorienting. In a similar paradigm, Bressler et al. [33] found that GC from parietal to occipital areas was predictive of behavioural performance. In a final and unusual example, Schippers et al. [34] used GC of fMRI signals to analyse directed interactions *between* the brains of two subjects engaged in a social game (charades), providing novel evidence for ‘mirror neuron system’ formulations of social interaction. Another promising application of GC is to intracranial local field potentials, which possess high temporal and spatial resolution and which comprise comparatively few variables (as compared to fMRI voxels or EEG sensors). An early application in this area, Gaillard et al. [35] examined directed functional connectivity during supraliminal as compared to subliminal visual word processing.

## Dynamic causal modelling

The basic idea behind DCM is that neural activity propagates through brain networks as in an input-state-output system, where causal interactions are mediated by unobservable (hidden) neuronal dynamics. This multi-input multi-output neuronal model is augmented with a forward, or observation model that describes the mapping from neural activity to observed responses. Together neuronal and observation model comprise a full generative model that takes a particular form depending on the data modality. The key outputs of DCM are the evidence for different models and the posterior parameter estimates of the (best) model, particularly those describing the coupling among brain regions. These allow for model and system identification, respectively.

DCM was introduced for fMRI timeseries [36], where the neuronal model comprises one or two hidden (lumped) neuronal states for each region. The neuronal dynamics of each region depend on the strength of connections within that region (parameterised by a self-connection), on the strength of external inputs (experimental input parameters) and on inputs from other regions in the network (the coupling parameters). Neuronal activity is then transformed through a haemodynamic model (with region-specific parameters) to model measured responses [37]. The coupling between brain regions can then be estimated for a particular model architecture using standard variational Bayesian techniques [36]. In practice, it is usual to specify different architectures or hypotheses and formally compare the evidence for these models, before examining parameter estimates [38]. DCM necessarily accounts for directed connections among brain regions and disambiguates the neuronal drivers of a particular event and subsequent signal propagation. Electrophysiological measurements support richer models of neuronal dynamics in DCM that comprise sources with laminar specific mixtures of neuronal populations. These have evolved from kernel-based models [7] that use postsynaptic convolution operators to describe responses at excitatory and inhibitory synapses to conductance-based models, where particular ion channels can be modelled and identified [39]. These neural mass models are accompanied by linear electromagnetic forward models to generate responses at EEG scalp electrodes, at MEG sensors or at intracranial recording sites. Application of DCM to animal local-field potential data has facilitated validation studies, where independent, invasive measurements (e.g. microdialysis or pharmacological perturbations) suggest that DCM can be used to estimate the physiological mechanisms responsible for mediating effective connectivity [40^•^].

## Pros and cons

Clearly, GC and DCM have complementary aims and strengths: GC can be applied directly to any given timeseries to detect the coupling among empirically sampled neuronal systems. This can provide useful insights into the system's dynamical behaviour in different conditions or in spontaneously active ‘resting’ states. One might then proceed to a more mechanistic (model or hypothesis — driven) characterisation using DCM. However, this calls for bespoke models of the system in question [41^••^]. In other words, GC is a generic inferential procedure characterising directed functional connectivity, while DCM is a framework that enforces (or enables) specific models or hypotheses to be tested. Crucially, both rest on model selection: In DCM this involves comparing the evidence for different models directly [38], while model selection in GC is implicit in the test for the presence of GC — and also arises in the selection of VAR model order, using standard approximations to model evidence, such as the Akaike or Bayesian information criteria [42].

Although GC is generic, its naive application is not always justified. For example, application to fMRI must recognise the indirect relation between neuronal activity and haemodynamic responses. In particular, regional variations in haemodynamic latency could confound the temporal precedence assumptions of GC [30]. While these variations can be partially controlled for by contrasting GC between experimental conditions [17, 43, 31] false inferences remain possible. Interestingly, recent theory and modelling suggests that GC may be robust to haemodynamic variations but not when combined with down-sampling and measurement noise [44]. In contrast, DCM models haemodynamic variations explicitly and tries to explain the data at the level of hidden neuronal states — in other words, it tries to get beneath the surface structure of the data to explain how they were generated: see [45, 46•] for further discussion.

In analysis of electrophysiological timeseries, GC is more widely accepted because there is no temporal lag between the responses recorded and their underlying (neuronal) causes and because the data can be sampled at fast timescales. The advantages of GC in furnishing frequency-dependent and multivariate measures have been clearly demonstrated [22, 26••, 28•]. However, there is an unresolved issue in this setting — the random fluctuations assumed by GC are serially independent (show no temporal correlations and fluctuate at very fast timescales). This is an issue because neuronal fluctuations in the brain are produced by neuronal systems that have the same time constants as the system studied. While serial independence can be checked for, the nature of neuronal fluctuations may deserve more attention in the future.

A key feature of DCM is that it can include variables that describe dynamics that are hidden from observation. For example, the GC analysis of anaesthetic loss of consciousness by Barrett et al. [28^•^] mentioned above, was complemented by a mechanistic study by Boly et al. [47^•^] using DCM. She found that a DCM that included a hidden thalamic source performed better than DCMs based solely on observed cortical timeseries, and established a dissociation between the effects of (measured) cortical and (inferred) subcortical structures on levels of consciousness. In contrast to GC, being able to model hidden sources means the model (hypothesis) space can be very large and calls for a principled approach to Bayesian model comparison of models that are (*a priori)* considered equally plausible. The specification and interrogation of the model space is an outstanding conceptual issue for DCM.

DCM posits and identifies neuronal mechanisms responsible for functional integration in the brain. Connectivity in this setting necessitates biologically plausible explanations. In DCM for fMRI, new developments [48, 49] enable the incorporation of background or ongoing spontaneous cortical fluctuations, nonlinearities and inhibitory neuronal populations [53]. The addition of spontaneous or stochastic fluctuations enhances the plausibility of the generative model at the neuronal level, where non-Markovian noise processes sit atop experimentally induced brain activations [48]. In DCM for electrophysiological data, the models will potentially allow the characterisation of receptor-specific contributions to brain connectivity, which may be important in a pharmacological and clinical setting [11^••^].

## Conclusion

In conclusion, GC and DCM are complementary: both model neural interactions and both are concerned with directed causal interactions. GC models dependency among observed responses, while DCM models coupling among the hidden states generating observations. Despite this fundamental difference, the two approaches may be converging. On the one hand DCM for stochastic systems [48] can now accommodate the random fluctuations assumed by GC. On the other hand, state-space GC approaches can incorporate modality specific observation equations [50]. The ability to handle large numbers of sources for regions is facilitated by multivariate (ensemble) GC [22] and sparse regression techniques [51], as well as recent developments in post hoc model optimisation for network discovery with DCM [52]. One might hope that both approaches — perhaps GC disclosing candidate models for DCM — will counter the claims that modern brain mapping is a neo-phrenology and provide characterisations of brain circuits that may hold promise for the treatment of neurological and psychiatric disorders.

## References and recommended reading

Papers of particular interest, published within the period of review, have been highlighted as:

• of special interest
•• of outstanding interest
